# Anaplastic lymphoma kinase-negative anaplastic large cell lymphoma with extranodal involvement of the thigh muscle: a case report

**DOI:** 10.1186/1752-1947-8-9

**Published:** 2014-01-06

**Authors:** Makoto Emori, Mitsunori Kaya, Shigeo Takahata, Hirotoshi Tobioka, Yasuhiko Minaki, Toshihiko Yamashita

**Affiliations:** 1 Department of Orthopedic Surgery, Sapporo Medical University School of Medicine, West 16, South 1, Chuo-ku, Sapporo, Hokkaido 060-8543, Japan; 2 Sapporo Maruyama Orthopedic Hospital, 27 Chome-1-3 North 7, Chuo-ku, Sapporo, Hokkaido 060-0007, Japan; 3 Division of Pathology, Otaru Kyokai Hospital, 1-6-15 Suminoe, Otaru, Hokkaido 047-8510, Japan; 4 Division of Orthopedic Surgery, Saiseikai Otaru Hospital, 10-1 Chikko, Otaru, Hokkaido 047-0008, Japan

**Keywords:** Anaplastic large cell lymphoma, Non-Hodgkin lymphoma, Extranodal involvement

## Abstract

**Introduction:**

Anaplastic large cell lymphoma is a rare type of non-Hodgkin lymphoma. The most common extranodal sites of anaplastic large cell lymphoma are skin, subcutaneous tissue, bone, lung, and gastrointestinal organs. The involvement of the skeletal muscle has been described rarely in extranodal anaplastic large cell lymphoma.

**Case presentation:**

An 89-year-old Japanese man visited our hospital with a three-month history of swelling of his left thigh and slight fever. The swelling had rapidly enlarged and become painful within the previous three days. Magnetic resonance imaging scans revealed two soft-tissue tumors in the intramuscular layer between the vastus medialis muscle and the adductor muscle. Extensive peritumoral inflammatory edema was obvious. As the results of physical and radiological examinations were highly suggestive of abscess formation, we prescribed antibiotics for two weeks. However, our patient’s symptoms did not improve. Therefore, we suspected a soft-tissue sarcoma, and our patient underwent an incision biopsy. Histological analysis revealed that the atypical cells were positive for CD3 and CD30 but negative for anaplastic lymphoma kinase. A computed tomography scan of the thorax revealed mediastinal lymphadenopathy and bilateral pleural effusions, suggestive of extranodal involvement of skeletal muscle in anaplastic lymphoma kinase-negative anaplastic large cell lymphoma. We planned to give our patient systemic chemotherapy. However, rapid systemic dissemination occurred and our patient died of multiple organ failure five weeks after his first visit to our hospital.

**Conclusions:**

Here, we present a case of anaplastic lymphoma kinase-negative anaplastic large cell lymphoma with extranodal involvement in the thigh muscle. The involvement of such a rare organ may lead to initial misdiagnosis and a delay in the onset of treatment.

## Introduction

Anaplastic large cell lymphoma (ALCL) is a subtype that represents 2 to 3% of non-Hodgkin lymphoma (NHL) [1]. The most common neoplasm metastases in skeletal muscle originate from carcinomas, leukemias, and lymphomas. Extranodal involvement of skeletal muscles in ALCL is extremely rare. To the best of our knowledge, this is the second reported case of anaplastic lymphoma kinase (ALK)-negative ALCL with extranodal involvement of the skeletal muscles [2]. In cases where multiple soft-tissue masses in the skeletal muscle are associated with fever and weight loss, extranodal involvement of the skeletal muscle should be a differential diagnosis.

## Case presentation

**Figure 1:**
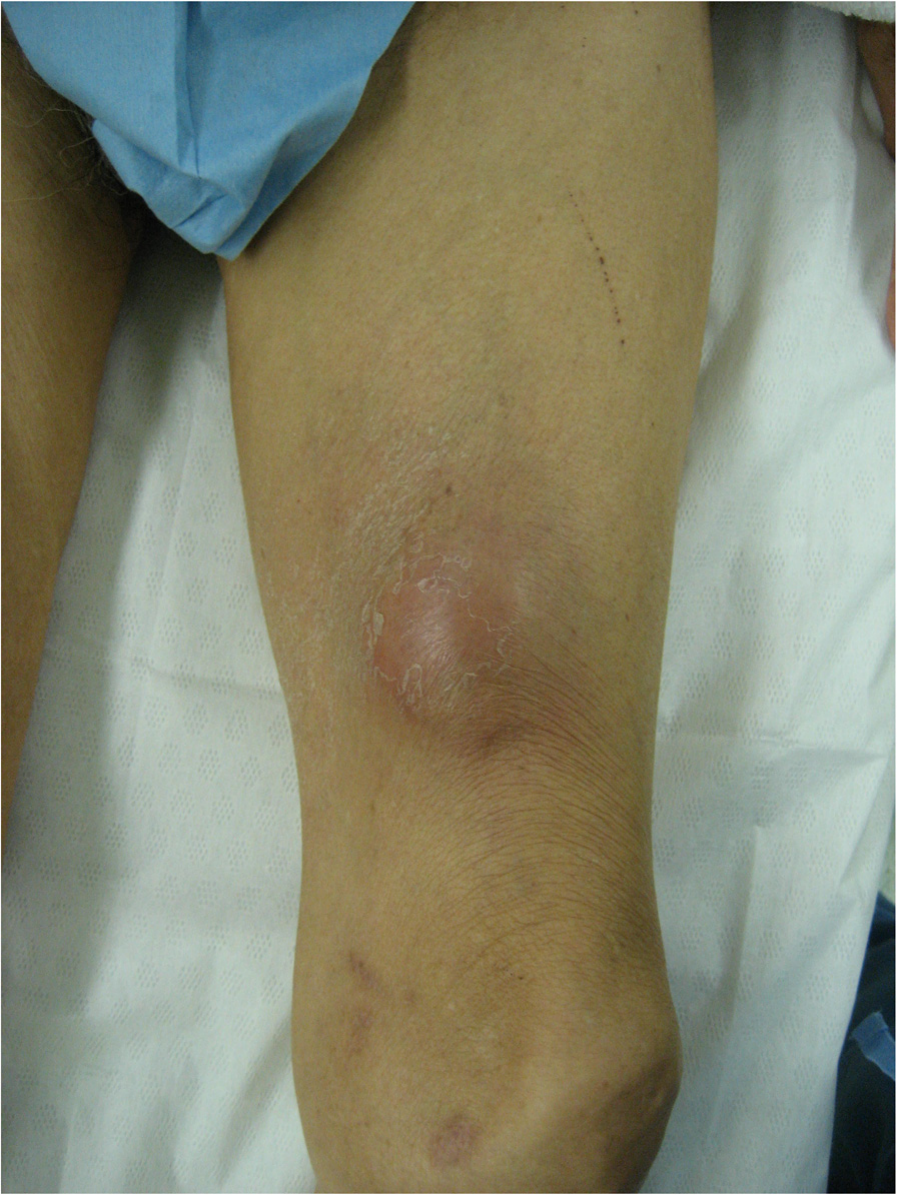
The tumor was elastic and soft with a relatively clear border, and located in the medial aspect of the left thigh.

**Figure 2:**
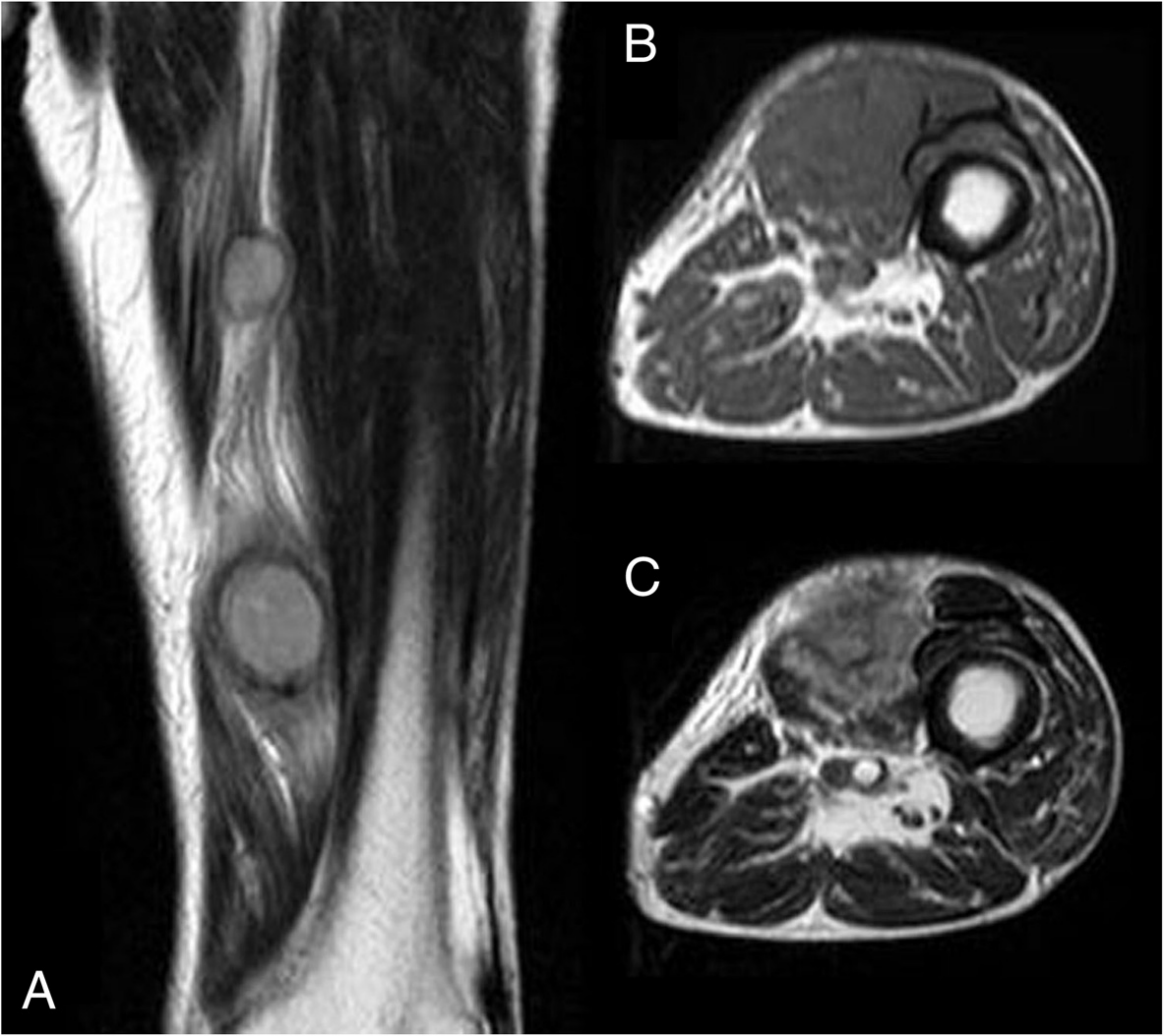
**Magnetic resonance imaging of the tumors. (A)** A T2-weighted coronal magnetic resonance imaging scan showed two soft-tissue tumors in the intramuscular layer between the vastus medialis muscle and the adductor muscle. Extensive peritumoral inflammatory edema was obvious. **(B)** A T1-weighted axial magnetic resonance imaging scan of the distal tumor (size, 4.0 × 4.0 × 3.5cm) showed homogeneous low signal intensity. **(C)** A T2-weighted axial magnetic resonance imaging scan showed heterogeneous low and high combined intensities.

**Figure 3:**
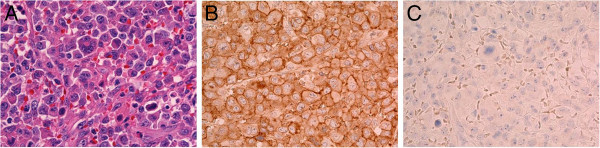
**Histological examination of the tumor. (A)** Histological examination of the biopsy sample showed dense infiltration of large, atypical, pleomorphic spindle cells with prominent nucleoli and mitotic figures. **(B, C)** Immunohistochemical examination revealed that the atypical cells were positive for CD30 **(B)**, but negative for anaplastic lymphoma kinase **(C)**.

An 89-year-old Japanese man visited our hospital with a three-month history of swelling of his left thigh and slight fever. The swelling had rapidly enlarged and become painful within the previous three days. The man had a one-month history of weight loss but other systemic symptoms or abnormal clinical findings, including night sweats, skin lesions, or swelling of the inguinal lymph nodes, were absent. A well-demarcated elastic soft mass (size, 7×6cm) was palpable in the medial aspect of the left thigh (Figure 1). Magnetic resonance imaging (MRI) scans revealed two soft-tissue tumors in the intramuscular layer between the vastus medialis muscle and the adductor muscle (Figure 2A). Extensive peritumoral inflammatory edema was obvious. T1-weighted axial imaging of the distal tumor (size, 4.0×4.0×3.5cm, Figure 2B) revealed homogeneous low signal intensity, whereas T2-weighted axial imaging (Figure 2C) revealed heterogeneous low and high combined intensities. The proximal tumor (size, 2.0×2.0×1.5cm), located 9cm proximal to the distal tumor, had the same intensity on the MRI scan (Figure 2A). Laboratory investigation showed an increased white blood cell count (10.9×10^9^ cells/L: neutrophils, 80%; lymphocytes, 18%; and monocytes 6%), erythrocyte sedimentation rate (100mm/h; normal value, <20mm/h), and C-reactive protein concentration (10.0mg/dL; normal value, <0.30ml/dL). Other biochemical and serologic parameters, including the lactate dehydrogenase level, were normal. As the results of physical and radiological examinations were highly suggestive of abscess formation, we prescribed antibiotics for two weeks. However, our patient’s symptoms did not improve. For pathological examination, we performed an open biopsy. Histological examination of the biopsy sample showed dense infiltration of large, atypical, pleomorphic spindle cells with prominent nucleoli and mitotic figures (Figure 3A, hematoxylin and eosin (H&E) stain, ×40). Immunohistochemical examination revealed that the atypical cells were positive for CD3, CD30 (Figure 3B, ×40), CD45, and CD45RO but negative for CD20, CD34, and ALK (Figure 3C, ×40). His human T-cell lymphotropic virus (HTLV) antibody result was negative. A computed tomography (CT) scan of the thorax revealed mediastinal lymphadenopathy and bilateral pleural effusions, suggestive of extranodal involvement of skeletal muscle in ALK-negative ALCL. His soluble interleukin-2 receptor (sIL2r) levels were extremely high, that is, 13800IU/mL (normal range, 220 to 530IU/mL). Therefore, we planned to give our patient systemic chemotherapy. However, rapid systemic dissemination occurred and our patient died of multiple organ failure five weeks after his first visit to our hospital. An autopsy was not performed.

## Discussion

ALCL was first described by Stein *et al.* in 1985 as pleomorphic large cell lymphoma with a strong expression of the cytokine receptor CD30 [3]. According to the fourth edition of the World Health Organization (WHO) classification, ALCL has two types of systemic ALCL: ALK-positive ALCL, and ALK-negative ALCL [4]. Subtyping based on the presence of the ALK protein is important because of the positive linkage between the presence of ALK and the prognosis of the patient [1,5]. ALK-positive disease has a more favorable prognosis with five-year survival as high as 80 to 90%, whereas ALK-negative ALCL is associated with advanced stage disease and has an overall five-year survival of approximately 40% [1]. Another main differentiating point between ALK-positive ALCL and ALK-negative ALCL is the difference in the age range affected. The most common extranodal sites are the skin, subcutaneous tissue, bone marrow, bone, lung, and gastrointestinal organs. In particular, lesions in the skeletal muscles are extremely rare in extranodal ALCL. To the best of our knowledge, this is only the second report of ALK-negative ALCL with extranodal involvement of the skeletal muscle [3].

The role of imaging studies in the diagnosis and treatment of muscle lymphomas has been discussed in various reports [6-8]. MRI is currently the most preferred method for the characterization of soft-tissue tumor. Lymphomas with muscle involvement will appear homogenous and isointense to muscle on T1-weighted MR images and homogenous and hyperintense with diffuse enhancement on T2-weighted MR images after intravenous administration of a contrast agent [6]. However, these MRI findings of tumors in muscles are not pathognomonic, and the differential diagnoses must include soft-tissue sarcoma, hematoma, and abscess [7]. When patients show multiple soft-tissue masses, clinicians should consider several possibilities like metastatic carcinoma in the skeletal muscle, purulent abscess, and malignant lymphoma. In this case, our patient did not have a history of cancer. Increased erythrocyte sedimentation rate and C-reactive protein levels and MRI findings of multiple soft-tissue masses with a peritumoral edematous reaction in the adjacent muscle led us to suspect abscess formation, causing a delay in diagnosis.

Patients with ALCL rarely present with multiple soft-tissue masses in the skeletal muscles. In the present case, our patient died five weeks after his first visit because of rapid systemic dissemination and this has also been reported earlier [5]. Therefore, the prognosis of ALK-negative ALCL with primary skeletal muscle involvement may be poorer than that of ALK-negative ALCL elsewhere. Clinicians are likely to encounter ALCL when patients present with multiple soft-tissue masses and B symptoms, that is, systemic symptoms of fever, night sweats, and weight loss. Therefore, careful observation will prevent the initial misdiagnosis and a delay in the onset of treatment.

## Conclusions

Here, we present a case of ALK-negative ALCL with extranodal involvement in the thigh muscle. The involvement of such a rare organ may lead to initial misdiagnosis and a delay in the onset of treatment.

## Consent

Written informed consent was obtained from the patient’s next of kin for publication of this case report and any accompanying images. A copy of the written consent is available for review by the Editor-in-Chief of this journal.

## Abbreviations

ALCL: Anaplastic large cell lymphoma; ALK: Anaplastic lymphoma kinase; NHL: Non-Hodgkin lymphoma.

## Competing interests

The authors declare that they have no competing financial interests.

## Authors’ contributions

ME assisted in the writing of the manuscript and in the orthopedic workup of the patient. MK assisted in the drafting of the manuscript. ST assisted in the writing of the manuscript and the orthopedic workup of the patient. YM assisted in the writing of the manuscript and the orthopedic workup of the patient. TY critically evaluated the manuscript and gave final approval for the manuscript to be published. All authors read and approved the final manuscript.
